# A Retrospective Cohort Study of Healthcare Utilization Associated with Paravertebral Blocks for Chronic Pain Management in Ontario

**DOI:** 10.1080/24740527.2021.1929883

**Published:** 2021-06-30

**Authors:** George Deng, Michael Gofeld, Jennifer N Reid, Blayne Welk, Anne MR Agur, Eldon Loh

**Affiliations:** a Vivo Cura Health, Calgary, Alberta, Canada; b Silver Pain Care, Toronto, Ontario, Canada; c ICES, Ontario, Canada; d Department of Surgery, Western University, London, Ontario, Canada; e Division of Anatomy, Department of Surgery, University of Toronto, Toronto, Ontario, Canada; f Department of Physical Medicine and Rehabilitation, Western University, London, Ontario, Canada; g Parkwood Institute Research, Lawson Health Research Institute, London, Ontario, Canada

**Keywords:** nerve blocks, pain management, interventional procedure, healthcare utilization

## Abstract

**Background**: Injections, particularly paravertebral blocks (PVBs), are frequently performed procedures in Ontario, Canada, for the management of chronic pain, despite limited evidence and risk of complications.

**Aim:** This study examines usage patterns of PVBs to evaluate their effects on healthcare utilization and opioid prescribing.

**Methods:** A retrospective cohort study in Ontario using administrative data. Ontario residents receiving their initial PVBs between July 1, 2013 and March 31, 2018 were included. Changes in use of other interventions, physician visits, and opioids were compared to the 12-month periods before and after index PVBs. Data use was authorized under section 45 of Ontario’s Personal Health Information Protection Act.

**Results:** 47,723 patients received their initial PVBs in the study period. The rate of index PVBs increased from 1.61 per 10,000 population (2013) to 2.26 per 10,000 (2018). Initial PVBs were performed most commonly by family physicians (*N* = 25,042), followed by anesthesiologists (*N* = 14,195). 23,386 patients (49%) received 1 to 9 repeat PVBs in the 12 months after index PVB; 12,474 patients (26.15%) received 10 or more. Use of other nonimage guided interventional pain procedures per patient (mean±SD) increased from 2.19 ± 9.35 to 31.68 ± 52.26 in the year before and after index PVB. Relevant physician visits per patient (mean±SD) also increased from 2.92 ± 3.61 to 9.64 ± 11.77. Mean opioid dosing did not change significantly between the year before and the year after index PVB.

**Conclusion:** PVBs are associated with increases in healthcare utilization and no change in opioid use patterns.

## RÉSUMÉ

**Contexte**: Les injections, en particulier les blocs paravertébraux (BPV), sont des procédures fréquemment effectuées en Ontario, Canada, pour la prise en charge de la douleur chronique, malgré des données probantes limitées et le risque de complications.

**Objectif**: Cette étude examine les modes d’utilisation des BPV afin d’évaluer leurs effets sur l’utilisation des soins de santé et la prescription d’opioïdes.

**Méthodes**: Étude de cohorte rétrospective utilisant les données administratives en Ontario. Les résidents de l’Ontario ayant reçu leur BPV initial entre le 1er juillet 2013 et le 31 mars 2018 ont été inclus. Les changements dans l’utilisation des autres interventions, les visites aux médecins et les opioïdes ont été comparés 12 mois avant et 12 mois après les BPV de référence. L’utilisation des données a été autorisée en vertu de la Loi sur la protection des renseignements personnels sur la santé de l’Ontario.

**Résultats**: 47 723 patients ont reçu leur BPV initial au cours de la période étudiée. Le taux de BPV est passé de 1,61 pour 10 000 habitants (2013) à 2,26 pour 10 000 (2018). Les BPV de référence étaient effectués le plus souvent par des médecins de famille (N = 25 042), suivis par les anesthésistes (N = 14 195). 23 386 patients (49 %) ont reçu de 1 à 9 BPV répétés dans les 12 mois suivant le BPV de référence ; 12 474 patients (26,15 %) en ont reçu 10 ou plus. L’utilisation d’autres procédures interventionnelles pour la douleur non guidées par l’image par patient (moyenne ± ET) est passée de 2,19 ± 9,35 à 31,68 ± 52,26 l’année précédant et suivant le BPV. Les visites médicales pertinentes par patient (moyenne ± ET) ont également augmenté de 2,92 ± 3,61 à 9,64 ± 11,77. La dose moyenne d’opioïdes n’a pas changé de manière significative entre l’année précédant et suivant le BPV de référence.

**Conclusion**: Les BPV sont associés à une augmentation de l’utilisation des soins de santé et ne sont associés à aucun changement dans les modes d’utilisation des opioïdes.

## Introduction

**Figure 1.**
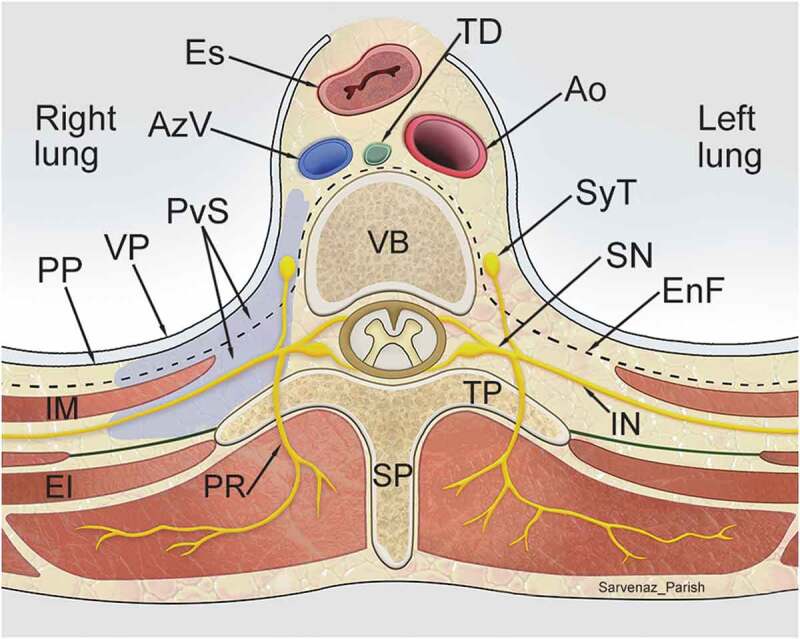
Transverse section showing extent and spatial relationships of the thoracic paravertebral space (purple) to surrounding structures. Nerves traversing through the paravertebral space, including the intercostal nerves and the sympathetic trunk, are targeted with paravertebral block. Ao, Aorta; AzV, Azygos vein; EI, External intercostal muscle; EnF, Endothoracic fascia; Es, Esophagus; IM, Innermost intercostal muscle; IN, Intercostal nerve; PP, Parietal pleura; PR, Posterior ramus; PvS, Paravertebral space; SN, Spinal nerve; SP, Spinous process; SyT, Sympathetic trunk; TD, Thoracic duct; TP, Transverse process; VP, Visceral pleura. (Illustration credit: Dr. Sarvenaz Parish)

“Nerve blocks” are a common, nonspecific term for chronic pain injection methods which involve instillation of local anesthetic (sometimes with the addition of corticosteroids) to temporarily interrupt the transmission of sensory information. They are a frequently performed procedure in Ontario pain clinics. A recent Toronto Star report has questioned the appropriateness of these blocks, highlighting a high frequency of injections, significant physician billings, and a lack of peer-reviewed evidence of efficacy.^1^ In 2012, the Ontario government unilaterally reduced reimbursement for paravertebral nerve blocks (PVBs), which represent a significant proportion of these injections. PVBs involve the infiltration of local anesthetic into the paravertebral compartment to temporarily anesthetize nearby neural structures (Fig. 1).^2^ In the pain literature, PVBs have only been evaluated in the context of acute trauma, cancer pain^3–6^ and perioperative regional anesthesia.^2,7–11^

Landmark techniques (compared to image-guided techniques) for PVBs have been shown to be unpredictable, with spread beyond the paravertebral space in 82% of cases^12^ and the potential for significant complications.^2,13–15^ Given the risks of PVBs, as well as the specific indications for these procedures, there is no obvious explanation accounting for the high prevalence of OHIP billings for PVBs. Despite criticism voiced by professional societies,^16^ some clinicians advocate for the effectiveness of PVBs, reporting pain reduction ≥50% in more than 70% of cases.^17^ Additionally, there are anecdotal patient reports that these injections are a better alternative to opioid use, and reduce healthcare utilization.^18,19^

The overall goal of this study is to assist healthcare providers and policy makers refine the appropriate use of PVB from a resource stewardship perspective, and to improve resource allocation for chronic pain management in Canada. By using a similar population-level methodology employed in our previous study evaluating the effectiveness of radiofrequency ablation in Ontario,^20^ the objectives of this retrospective cohort study were to: (1) explore the patient and healthcare utilization characteristics associated with the use of PVBs in Ontario, and (2) to evaluate the impact of PVBs on healthcare utilization and opioid use in patients with chronic pain receiving these interventions in Ontario.

## Methods

### Design and Setting

A retrospective cohort study was conducted using administrative data from the province of Ontario, Canada. The study included residents of Ontario who received their initial PVB between July 1, 2013 and March 31, 2018. Since the Narcotics Monitoring System was not available until 2013, that is when the study period began. The end date was chosen to allow a sufficient follow-up period to evaluate outcomes. Data use in this project was authorized under section 45 of Ontario’s Personal Health Information Protection Act, which does not require review by a Research Ethics Board. Individual patient consent was not required.

#### Data Sources

Several linked administrative data sources were used for this study, including: (1) Narcotics Monitoring System (NMS, containing all opioids dispensed in the province); (2) Canadian Institute for Health Information Discharge Abstract Database/Same Day Surgery (CIHI-DAD/SDS, identifying all hospital admissions and procedures); (3) National Ambulatory Care Reporting System (NACRS, identifying all emergency room encounters); (4) Ontario Health Insurance Plan (OHIP, containing all physician billing codes for patient assessment or treatment); (5) ICES physician database alongside (6) Corporate provider database (IPDB, CPDB; (identifying physician specialty and years of practice); (7) Registered Persons database (RPDB, containing vital statistics and patient demographics); (8) Patient Contact and Eligibility Yearly Files (CONTACT, identifying prior patient healthcare contact information); and (9) Ontario Population Estimates and Projections, distributed by the Ontario Ministry of Health and Long-Term Care: IntelliHEALTH Ontario (POP, used to calculate rates of PVB procedures over time). Data was analyzed at ICES Western using unique, encoded personal identifiers to link datasets. The validity of the data elements in the above databases has been previously documented.^21–25^

#### Patient Population

Patients with PVB performed between July 1, 2013 and March 31, 2018 were identified by the unique physician OHIP billing codes G228 (billing for the first PVB) and G123 (billing for subsequent PVB on the same clinical encounter). The first occurrence of the PVB OHIP billing code was considered the index date.

Exclusion criteria for the main cohort included: PVB in the 5 years prior to the index date (to ensure index treatments were identified), no healthcare contact between 3 to 5 years prior to the index date (to ensure patients would have a minimum 2-year administrative data history), nonresidents of Ontario, patients who died or emigrated from the province during the 1-year period after the index date, and PVBs done on Emergency room visits.

#### Outcomes

The primary outcome was the change in healthcare and opioid utilization in the immediate 12-month periods before and after the index date. Healthcare utilization was assessed through changes in (1) overall physician visits, (2) physician visits in Family Medicine, Physical Medicine and Rehabilitation (PMR), Orthopedics, Neurology, Neurosurgery, or Anesthesiology, and (3) the use of interventional pain procedures other than PVB. Utilization characteristics of image-guided procedures, versus those that do not require imaging, were also evaluated. Image-guided interventional procedures were defined as those procedures whose OHIP codes require the use and documentation of image-guidance to submit a billing. These codes included fluoroscopic-guided cervical/thoracic/lumbar facet intra-articular injection/medial branch blocks, ultrasound-guided lumbar medial branch blocks, fluoroscopic-guided sacroiliac joint injections, fluoroscopic stellate ganglion blocks, and nerve root injections. Opioid utilization was assessed by comparing (1) total daily oral morphine equivalent (MEQ) dosing, and (2) total number of opioid prescriptions, in the year before and after the index date.

Secondary outcomes were the number of each specialty billing for these injections, the proportion of people who received multiple PVBs in a year, the average number of PVBs per person performed in a year, the average time between subsequent PVBs, and the number of levels billed per PVB. The number of emergency room visits, and imaging requirements (e.g., CT/MRI), in the 2 week period post-procedure was used to estimate adverse events associated with PVBs.

#### Statistical Analysis

Differences between pre-post PVB groups was tested using a Wilcoxon signed-rank test. In all pre-post group comparisons, a two-sided *p*-value <0.05 was considered significant. A test for trend on the rate of index PVB procedures between July 1, 2013 and March 31, 2018 was carried out using linear regression, where a two-sided *p*-value <0.05 was considered significant. In SAS,^26^ PROC REG was used, and model assumptions of homoscedasticity and normally distributed residuals were confirmed graphically. Additionally, no multicollinearity was confirmed using VIF, and Cook’s D statistic ensured no unusual outliers. Descriptive statistics were used to describe distributions of the pre-post PVB groups. All analyses were conducted using SAS version 9.4 (SAS Institute, Cary, NC). In accordance with ICES privacy policies, cell sizes less than or equal to five cannot be reported.

## Results

**Figure 2.**
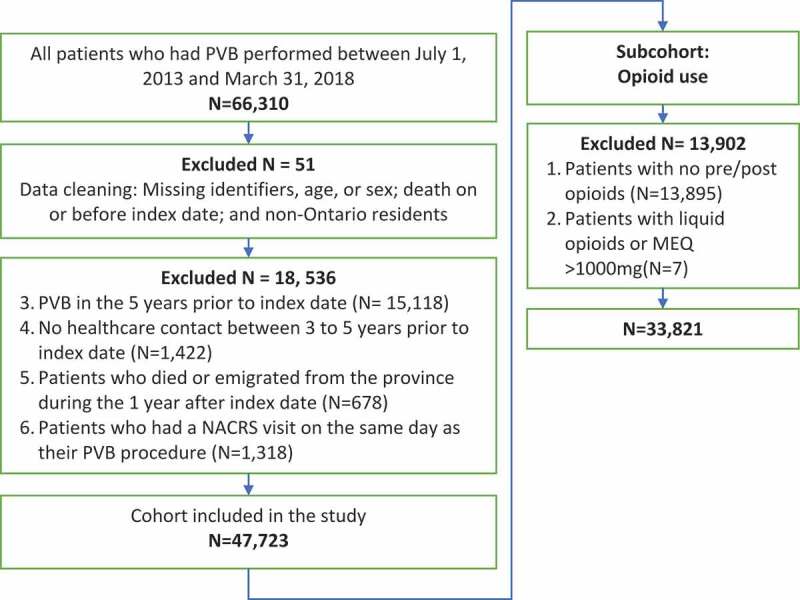
A flowchart outlining overall cohort size and opioid analysis subcohort determination. NACRS, National Ambulatory Care Reporting System; PVB, paravertebral block; MEQ, morphine equivalents

A total of 47,723 patients between July 1, 2013 and March 31, 2018 met inclusion criteria (Fig. 2). 60.42% were female (*N = *28,832) and mean age was 51.34 years (standard deviation (SD)±15.72 years). On a per capita basis, using the POP dataset, the rate of index PVBs increased from 1.61 per 10000 population in the first study period (third quarter of 2013) to 2.26 per 10,000 in the final study period (first quarter of 2018) (*p* < .0001).

On the index date, 11.99% (*N* = 5,720) had 1 PVB level billed, and 87.99% (*N* = 41,992) had 2 levels billed (mean±SD; 1.88 ± 0.33). PVBs were most prevalently performed by family physicians (52.47%) followed by anesthesiologists (29.74%) (Table 1).

**Table 1.** Number of index PVBs performed by specialty in Ontario, Canada

Specialty	Index PVB, *N*(%) patients
Family Medicine	25,042 (52.47%)
Anesthesiology	14,195 (29.74%)
Radiology	2,280 (4.78%)
ER	1,743 (3.65%)
PMR	1,352 (2.83%)
Orthopedic	1,172 (2.46%)
Neurosurgery	1,034 (2.17%)
Other	779 (1.63%)
Neurology	126 (0.26%)

On the day of the index PVB, 85.43% (*N* = 40,768) of all patients received at least one additional injection (mean±SD; 2.42 ± 1.58 additional injections). The most commonly billed additional injection on the index date was trigger point injection (Table 2). A total of 1,606 patients (3.37%) visited an emergency room within the first 14 days post-PVB and a total of 992 patients (2.08%) had a CT and/or MRI within the first 14 days post-procedure.

**Table 2.** The most common procedures/nerve blocks performed on the same day as the index PVB and the number/proportion of patients receiving each procedure listed in descending order

Types of Procedure/Nerve Block Performed on Same Day as Index PVB	Number (%) of Patients
Trigger point	25,821 (54.11%)
Injection of bursa, or injection/aspiration of joint, ganglion, or tendon sheath	25,380 (53.18%)
Scapular nerve	14,227 (29.81%)
Other cranial nerves	13,171 (27.60%)
Sciatic	11,727 (24.57%)

### Healthcare Utilization before and after Index Date

#### PVB and Other Interventional Procedures

In the year following the index PVB, 49% of patients received between 1 and 9 repeat PVBs, 26.15% of patients received 10 or more repeat PVBs and 24.86% did not have follow-up PVB. 7.77% of the sample (N = 3,706) received 30 or more repeat PVBs during the year (supplemental material, Appendix 1). The average (± SD) number of days between subsequent PVBs was 32.38 ± 44.66.

The mean (± SD) number of other interventional pain procedures received per patient increased from 2.19 ± 9.35 in the year before index PVB to 31.68 ± 52.26 the year after. 49.3% of the cohort received 10 or more other interventional pain procedures in the post-period (supplemental material, Appendix 2). Of patients who had at least one interventional procedure prior to index PVB, there was a significant increase in the total number of procedures (excluding specific image-guided procedures) in the pre- to post-period, from 104,641 to 238,984 (*p* < .0001). In this group, the most commonly performed interventional procedures in the post-period was injection of bursa/joint/ganglion tendon sheath (Table 3; supplemental material Appendix 3).

**Table 3.** The five most common interventional procedures (other than PVB and certain specific image-guided procedures) performed in the year after the index PVB. This comparison is done on a subgroup of patients who had at least one interventional procedure prior to index PVB. *indicates a statistically significant change (*p* < .0001) from the year before to the year after

Type of Procedure Performed	Procedures Performed in Pre-Period (*N*, total procedures)	Procedures Performed in Post-Period (*N*, total procedures)
Injection of bursa, or injection/aspiration of joint, ganglion, or tendon sheath	20,019	71,035*
Trigger point	17,585	46,877*
Scapular nerve	9,700	31,389*
Other cranial nerves	8,461	28,192*
Intramuscular, subcutaneous or transdermal injection	16,539	12,659*

Interventional procedures requiring image guidance for OHIP billing, such as medial branch blocks, were performed in only 2.9% of the total cohort in the pre-period and 7.4% of the cohort in the post-period. In contrast to interventional procedures that do not require imaging, <1% received 5 or more image-guided procedures in the year after the index event (supplemental material, Appendix 4). Additionally, while the number of patients receiving an image guided injection in the post-period increased, the total number of procedures decreased.

### Physician Visits

The mean ± SD number of physician visits to indexed specialites per patient increased from 2.92 ± 3.61 to 9.64 ± 11.77 in the 1 year pre- to 1 year post-period (Table 4). Of those with at least one physician visit prior to the index PVB, total number of physician visits increased from 139,559 in the pre-period to 308,541 in the post-period (*p* < .0001) (Table 4). The greatest increase related to family medicine visits. The post-period increase in visits relates primarily to PVB injections; when these visits are excluded there is only a slight post-period increase in total physician visits (Table 4). There was a reduction of visits to orthopedic, neurology, and physical medicine and rehabilitation specialists and a small increase in visits to neurosurgery (Table 4).

**Table 4.** Total physician visits and mean physician visits with standard deviation (SD) per patient by specialty before and after index PVB, including and excluding visits related to PVB. PVB, paravertebral block. The comparison of total physician visits is done on a subgroup of patients who had at least one physician visit prior to index PVB. *indicates a statistically significant change (*p* < .0001)

	Total Physician Visits		Total Visits per Patient		Total Visits per Patient Excluding PVB	
	Pre-Period (*N*, total visits)	Post-Period (*N*, total visits)	Pre-Period (Mean ± SD)	Post-Period (Mean ± SD)	Pre-Period (Mean ± SD)	Post-Period (Mean ± SD)
Total number of relevant physician visits	139,559	308,541*	2.92 ± 3.61	9.64 ± 11.77	2.92 ± 3.61	3.71 ± 5.28
Family Medicine visits	62,507	212,214*	1.31 ± 2.65	6.06 ± 11.01	1.31 ± 2.65	1.82 ± 4.36
Orthopedics visits	25,749	20,048*	0.54 ± 1.34	0.72 ± 2.44	0.54 ± 1.34	0.52 ± 1.35
Anesthesia visits	22,997	54,990*	0.48 ± 1.47	1.95 ± 4.72	0.48 ± 1.47	0.82 ± 2.31
Neurology visits	13,951	6,879*	0.29 ± 0.85	0.27 ± 0.89	0.29 ± 0.85	0.25 ± 0.79
Physical Medicine and Rehabilitation visits	10,044	5,695*	0.21 ± 0.80	0.25 ± 1.09	0.21 ± 0.80	0.20 ± 0.83
Neurosurgery visits	4,311	8,715*	0.09 ± 0.56	0.39 ± 3.07	0.09 ± 0.56	0.10 ± 0.62

### Opioid Utilization

**Figure 3.**
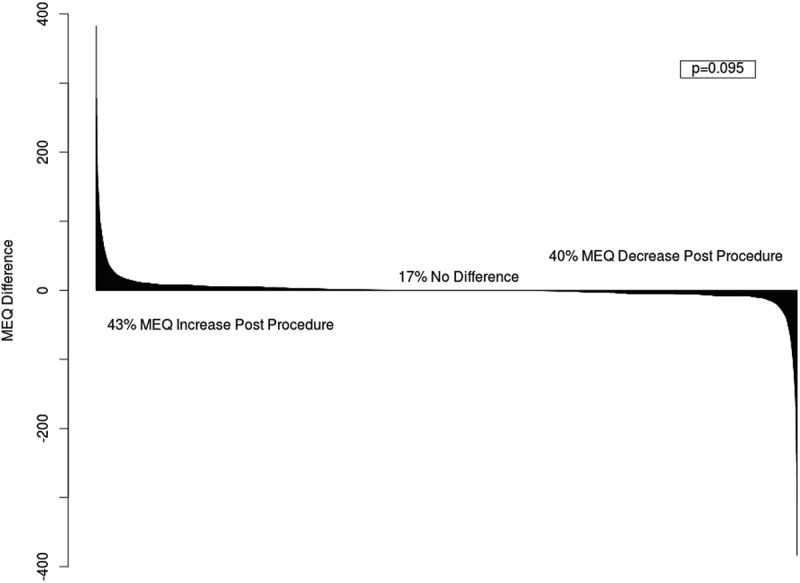
Waterfall plot of differences in total daily oral morphine equivalent (MEQ) dosing 1 year pre-PVB and post-PVB

A total of 33,821 patients met the inclusion criteria for this analysis (Fig. 2). In the year before and after the index PVB, the mean MEQ ± SD was 13.01 ± 32.03 mg and 13.17 ± 30.61 mg respectively. Overall, 43% had an increase, 17% had no change, and 40% had a decrease in MEQ dosing post-PVB (*p* = .095) (Fig. 3).

There were 28,807 patients who received an opioid prescription in the year prior to their index PVB. Of those patients, 7,007 patients did not receive any opioid prescriptions in the year after the index PVB. The majority of those who did not require opioid prescriptions in the post-period had only 1 prescription in the pre-index PVB period (Table 5).

**Table 5.** Prescriptions provided to patients before and after index PVB, in the cohort who had at least one opioid prescription in the pre-period (*N* = 28,807)

Number of Prescriptions	Pre-Period (*N*, patients)	Post-Period (*N*, patients)
**0**	0	7,007
**1**	6,521	2,942
**2**	3,407	1,894
**3**	2,330	1,391
**4**	1,705	1,200
**≥5**	14,844	14,373

## Discussion

Significant healthcare and societal resources are dedicated to chronic pain management, and consequences of untreated pain are dire.^27–29^ The results of this study raise concerns regarding use of PVBs. First, PVBs are frequently billed once treatment is initiated. Second, PVBs are associated with increases in healthcare utilization, as measured by physician visits and other interventions. Third, there is minimal change in opioid utilization post-treatment.

### Healthcare Utilization – Interventional Procedures

Many patients in this study received multiple concurrent injections after PVB was initiated. Although patients may report anecdotal benefit from this combined treatment regimen, the evidence that these procedures provide significant pain relief is limited.^30–33,34,35^

The need for multiple different regional procedures suggests that these patients have fairly diffuse pain. Central sensitization and psychosocial factors may better account for this type of pain; the ability of multiple injections to provide meaningful relief in this scenario is questionable. Alternative hypotheses for benefit include placebo response, positive therapeutic alliance from frequent visits, and provider expectations.

The paucity of image-guided procedures performed in this cohort may reflect the need for specialized equipment and training, and better-defined selection criteria for these procedures. Interestingly, there was a significant decrease in the number of neuraxial interventional procedures following index PVB (Appendix 3), whereas there was a 3.5 fold increase in injection of bursa, or injection/aspiration of joint, ganglion, or tendon sheath; trigger point injection; scapular nerve block; and other cranial nerves block (Table 3). This drastic change in management approach after initiation of PVB is unusual, as pain symptoms and signs are not expected to significantly change following PVB. The question arises as to whether the change in procedural billing patterns post-index PVB is driven by other factors, including financial considerations.

### Healthcare Utilization – Physician Visits

Family physicians had the highest increase in physician visits post-index PVB, followed by anesthetists. The increase in visits post-PVB was driven mainly by repeated PVB injections, as physician visits only slightly increased when PVB visits were excluded. The high incidence of family physician PVB billings is unexpected, as indications for PVB are highly specific and would likely be outside the scope of practice for most family physicians.

One possible explanation for the high frequency of these billings is that other nonimage guided interventional procedures (e.g., trigger point injections) are being misclassified as PVBs. For example, a physician may bill insertion of needles into the paraspinal musculature (e.g., multifidus, erector spinae) as a PVB, although a trigger point injection may be a more accurate description. Even within the literature, “paravertebral injection” has been used to describe procedures such as facet joint injections and/or medial branch blocks.^14,15^ It is possible that PVB and other nonimage guided interventional codes in Ontario may be used in place of more appropriate billing codes that are less remunerative or have more restrictive conditions on their use.

The high number of procedures performed suggests that this is not an issue involving a small group of physicians but a wider systemic issue. The collective billing of PVB and associated interventional procedures have totaled over 420 million CAD since 2011,^1^ with physicians billing multiple millions per year for these injections.^19^ Although a discussion on billing and resource stewardship ethics is beyond the scope of this article, the study team notes that allocating funds to PVB and associated procedures may decrease access to other options for pain management, and may affect the availability of healthcare resources generally (i.e., nonpain management resources). Ongoing education on appropriate indications for pain interventions may improve not only healthcare utilization but also patient outcomes.

The study team notes that there is a reduction in visits to some specialties following initiation of PVBs, specifically orthopedics, PMR, and neurology. There may be several reasons for this decrease, but improvement in patient symptoms that negates the need to involve these specialties cannot be excluded.

### Opioid Utilization

A proportion of those who received opioid prescriptions in the year prior to PVB did not require one in the year after. However, most of these patients only had one prescription in the year pre-procedure. Thus, their opioid use may not have been high to begin with, or may have been incidental and not related to the indication for PVB. In contrast, the majority of those who received five or more prescriptions in the year before PVB continued to receive a similar number of prescriptions in the year following. Mean opioid dose did not significantly change in the year before and after index PVB; the overall effect of PVB and associated interventions on opioid utilization is uncertain.

### Comparison to Radiofrequency Ablation

In Ontario, radiofrequency ablation (RFA) and its impact on healthcare utilization, using the same outcome measures, has also been assessed using population level data.^20^ Although a formal control group was not included in this paper, the results from the RFA paper can be useful in contrasting the outcomes of this paper. In contrast to PVB, the mean (± SD) time between repeat RFA was much longer (432.20 ± 375.28 days) compared to PVB (32.38 ± 44.66 days). Importantly, RFA significantly reduced healthcare utilization in the year post-procedure in terms of physician visits and repeat interventional procedures. Based on this data and the existing literature,^36–38^ there is still an important role for targeted interventions that are appropriately applied.

### Risks

PVBs are associated with a number of potential risks, including vascular puncture, pneumothorax, pleural puncture, hematoma, signs of epidural or intradural spread, pain at injection site, symptomatic bradycardia and hypotension, vasovagal response, and local anesthetic toxicity.^2,13–15^ The authors note that ED visits and the need for imaging after PVB is similar to that after the Ontario RFA study^20^ (ED visits: 3.25% (RFA) vs. 3.37% (PVB); imaging studies: 1.68% (RFA) vs. 2.08% (PVB)), although the follow-up inclusion period for ED visits and imaging studies was longer in the RFA study (up to 4 weeks compared to only 2 weeks in this study). While emergency department visits and the need for further advanced imaging modalities was used as a surrogate to identify any potential risks, it cannot be assumed that these visits/imaging studies are necessarily related to the procedure themselves.

### Limitations

A limitation of this study is that it is retrospective, and misclassification errors may occur within the provincial database. The exact indication for PVBs was unknown as the OHIP billing code does not discriminate between chronic pain versus other reasons for PVB, so it is possible that the high proportion of PVBs in Ontario is due to a reason other than chronic pain. However, the authors find it less likely that the large proportion of PVBs in Ontario relate to nonchronic pain indications for two reasons. First, specific indications for PVBs in the literature (e.g., acute trauma, cancer pain^3–6^ and perioperative regional anesthesia^2,7–11^) imply time-limited treatments in highly specialized environments. However, we found that the majority of billing instances of these procedures involve more generalized practitioners (e.g., Family Medicine) and occur recurrently over a prolonged period of time. Second, we excluded all patients who had a NACRS visit on the same day as their index PVB (*n* = 1318). NACRS includes all emergency department visits, which the authors reasoned would help to eliminate those receiving PVBs for other reasons (e.g., trauma).

Another major limitation of the study was a lack of a formal control group. The study team was able to make comparisons to a previous study on RFA in Ontario, which utilized a similar methodology and assessed similar outcomes. However, the current study did not specifically include a control group where no interventional pain procedures were provided, which could affect interpretation of the findings. We note that it would be difficult to identify such a control group because there are no specific pain assessment OHIP billing codes available; OHIP billing codes are specialty provider specific (with no specific codes within Pain Medicine outside of procedural codes).

The outcome measures that can be assessed are limited as a result of the use of an administrative database. Changes in outcomes such as pain, sleep, function, and mood are not quantifiable given the nature of the data collected within the available databases. Other measures not available within administrative databases include allied health access and changes in employment status (i.e., return to work). Additionally, we did not quantify the use of over the counter medications, illicit drug use, cannabis, and pain modulating antidepressants and antiepileptics.

Data was interpreted using PVBs as the index procedure due to its high yearly cumulative billing. However, the authors suspect that using another nonimage guided interventional procedure as the index would yield similar findings.

Conclusions regarding opioids were more difficult to interpret as there have been efforts to reduce opioid prescription in chronic pain patients during the study period.

## Conclusion

PVBs for pain management were associated with increased total physician visits, increased use of other nonimage-guided interventional procedures, and uncertain effects on opioid utilization. A broader discussion on the ongoing, prevalent use of this modality for chronic pain management, particularly given a lack of evidence in the literature assessing its effectiveness, should be considered.

## Supplementary Material

Supplemental Material
