# Supplementary material for: A Retrospective Cohort Study of Healthcare Utilization Associated with Paravertebral Blocks for Chronic Pain Management in Ontario
Source: Can J Pain. 2021 Jun 30;5(1):130–8. doi: 10.1080/24740527.2021.1929883 (PMC8253135; doi:10.1080/24740527.2021.1929883)
Supplement: Supplemental Material [file UCJP_A_1929883_SM2068.zip › Appendix 1.docx]

| Number of repeat PVB | Number (%) of patients |
| --- | --- |
| 0 | 11,863 (24.86%) |
| 1 | 6,226 (13.05%) |
| 2 | 4,664 (9.77%) |
| 3 | 3,211 (6.73%) |
| 4 | 2,412 (5.05%) |
| 5 | 1,999 (4.19%) |
| 6 | 1,546 (3.24%) |
| 7 | 1,306 (2.74%) |
| 8 | 1,094 (2.29%) |
| 9 | 928 (1.94%) |
| 10-19 | 5,691 (11.93%) |
| 20-29 | 3,077 (6.45%) |
| 30-39 | 1,794 (3.76%) |
| 40-49 | 1,592 (3.34%) |
| 50-59 | 267 (0.56%) |
| >=60 | 53 (0.11%) |

Appendix 1: Number and proportion of patients in the overall cohort that received a specific number of repeat PVB in the year following the index date.
