# Supplementary material for: A Retrospective Cohort Study of Healthcare Utilization Associated with Paravertebral Blocks for Chronic Pain Management in Ontario
Source: Can J Pain. 2021 Jun 30;5(1):130–8. doi: 10.1080/24740527.2021.1929883 (PMC8253135; doi:10.1080/24740527.2021.1929883)
Supplement: Supplemental Material [file UCJP_A_1929883_SM2068.zip › Appendix 2 JR.docx]

| **Number of Procedures** | **Pre-period (N (%), patients)** | **Post-period (N (%), patients)** |
| --- | --- | --- |
| **0** | 29,956 (62.8%) | 8,466 (17.7%) |
| **1** | 5,686 (11.9%) | 2,392 (5.0%) |
| **2** | 3,365 (7.1%) | 2,483 (5.2%) |
| **3** | 2,049 (4.3%) | 2,189 (4.6%) |
| **4** | 1,467 (3.1%) | 2,072 (4.3%) |
| **5** | 925 (1.9%) | 1,429 (3.0%) |
| **6** | 757 (1.6%) | 1,695 (3.6%) |
| **7** | 518 (1.1%) | 972 (2.0%) |
| **8** | 423 (0.9%) | 1,457 (3.1%) |
| **9** | 317 (0.7%) | 1,062 (2.2%) |
| **>=10** | 2,260 (4.7%) | 23,506 (49.3%) |

Appendix 2. Number and proportion of patients in the overall cohort (n=47,723) that received a specific number of other interventional procedures (excluding specific image-guided procedures) in the year before and after the index date.
