# Supplementary material for: A Retrospective Cohort Study of Healthcare Utilization Associated with Paravertebral Blocks for Chronic Pain Management in Ontario
Source: Can J Pain. 2021 Jun 30;5(1):130–8. doi: 10.1080/24740527.2021.1929883 (PMC8253135; doi:10.1080/24740527.2021.1929883)
Supplement: Supplemental Material [file UCJP_A_1929883_SM2068.zip › Appendix 3 JR.docx]

| **Type of Procedure Performed** | **Procedures Performed in Pre Period (N, procedures)** | **Procedures Performed in Post Period (N, procedures)** |
| --- | --- | --- |
| Injection of bursa, or injection/aspiration of joint, ganglion, or tendon sheath | 20,019 | 71,035* |
| Trigger point | 17,585 | 46,877* |
| Scapular nerve | 9,700 | 31,389* |
| Other cranial nerves | 8,461 | 28,192* |
| Intramuscular, subcutaneous or transdermal injection | 16,539 | 12,659* |
| Not listed nerve | 5,207 | 10,708* |
| Sciatic | 2,641 | 7,598* |
| Occipital nerve | 4,656 | 6,321 |
| Trigeminal/mandibular nerve | 2,992 | 5,770* |
| Supraorbital | 2,741 | 5,358* |
| Lumbar epidural injections | 4,749 | 3,044* |
| Brachial plexus | 1,127 | 2,466* |
| Pudendal | 850 | 2,159* |
| Ilioinguinal and iliohypogastric | 720 | 1,092 |
| Infraorbital | 708 | 1,041 |
| Femoral nerve | 651 | 1,026 |
| Caudal epidural injections | 979 | 663* |
| Aspiration of bursa or complex joint, with or without injection | 1,369 | 525* |
| Intercostal | 285 | 279 |
| Obturator | 119 | 216 |
| Cervical epidural injections | 413 | 186* |
| Peripheral nerve block, major (acute) | 460 | 120* |
| Major plexus block (acute) | 388 | 64* |
| Peripheral nerve block, minor (acute) | 416 | 62* |
| Thoracic epidural injections | 105 | 56 |
| Epidural/spinal injection of narcotic (acute) | 610 | 52* |
| Sympathetic nerve injections | 47 | 22 |
| **TOTAL** | **104,641** | **238,984*** |

Appendix 3. The number of interventional procedures (other than PVB and certain specific image-guided procedures) performed in the year before and after the index PVB. This comparison is done on a subgroup of patients who had at least one interventional procedure prior to index PVB. Laryngeal nerve, Caudal, single injection (acute), and Thoracic epidural with catheter (acute) were included in total counts; however, due to frequencies less than or equal to five, numbers could not be reported in accordance with ICES privacy policies.* indicates a statistically significant change (p<0.0001).
