# Supplementary material for: A Retrospective Cohort Study of Healthcare Utilization Associated with Paravertebral Blocks for Chronic Pain Management in Ontario
Source: Can J Pain. 2021 Jun 30;5(1):130–8. doi: 10.1080/24740527.2021.1929883 (PMC8253135; doi:10.1080/24740527.2021.1929883)
Supplement: Supplemental Material [file UCJP_A_1929883_SM2068.zip › Appendix 4 JR.docx]

| **Number of Procedures** | **Pre-Period, N (%) patients** | **Post-Period, N (%) patients** |
| --- | --- | --- |
| **0** | 46,340 (97.1%) | 44,203 (92.6%) |
| **1** | 619 (1.3%) | 1,467 (3.1%) |
| **2** | 419 (0.9%) | 973 (2.0%) |
| **3** | 168 (0.4%) | 386 (0.8%) |
| **4** | 91 (0.2%) | 290 (0.6%) |
| **5** | 41 (0.1%) | 115 (0.2%) |
| **6** | 18 (0.0%) | 94 (0.2%) |
| **7** | 10 (0.0%) | 46 (0.1%) |
| **8** | <=10 | 37 (0.1%) |
| **9** | <=5 | 19 (0.0%) |
| **>=10** | 9 (0.0%) | 93 (0.2%) |

Appendix 4. Number and proportion of patients in the overall cohort (n=47,723) that received a specific number of image-guided interventional procedures in the year before and after the index date. In accordance with ICES privacy policies, cell sizes less than or equal to five cannot be reported.
